# Advancing Drug Discovery through Academia–Industry Collaborations: Lessons from Merck Discovery Chemistry Partnerships

**DOI:** 10.1021/acscentsci.6c00774

**Published:** 2026-07-31

**Authors:** Beryl X. Li, Dipannita Kalyani

**Affiliations:** † Discovery Chemistry, 2793 Merck & Co., Inc., South San Francisco, California 94080, United States; ‡ Discovery Chemistry, Merck & Co., Inc., Rahway, New Jersey 07065, United States

## Abstract

Academia–industry partnerships are an important driver of innovation in drug discovery, and their success depends on intentional problem selection, effective resource sharing, and robust communication during project execution. This outlook highlights select Merck Sharp & Dohme Discovery Chemistry (MSD DC) collaborations reported over the past five years to illustrate principles that underpin productive academia–industry partnerships. Through representative case studies in synthesis collaborations, we illustrate two complementary collaboration strategies: targeted development of synthetic methods that address key medicinal chemistry bottlenecks, and capability-building efforts that enable technology transfer to accelerate drug discovery. We also highlight a few nonsynthetic collaborations by MSD DC in computational chemistry, structure elucidation, and chemical biology subfields, underscoring the cross-functional impact of these efforts on drug discovery. Beyond scientific advances, these collaborations also provide meaningful training and professional development opportunities for the next generation of chemists.

## Graphical abstract

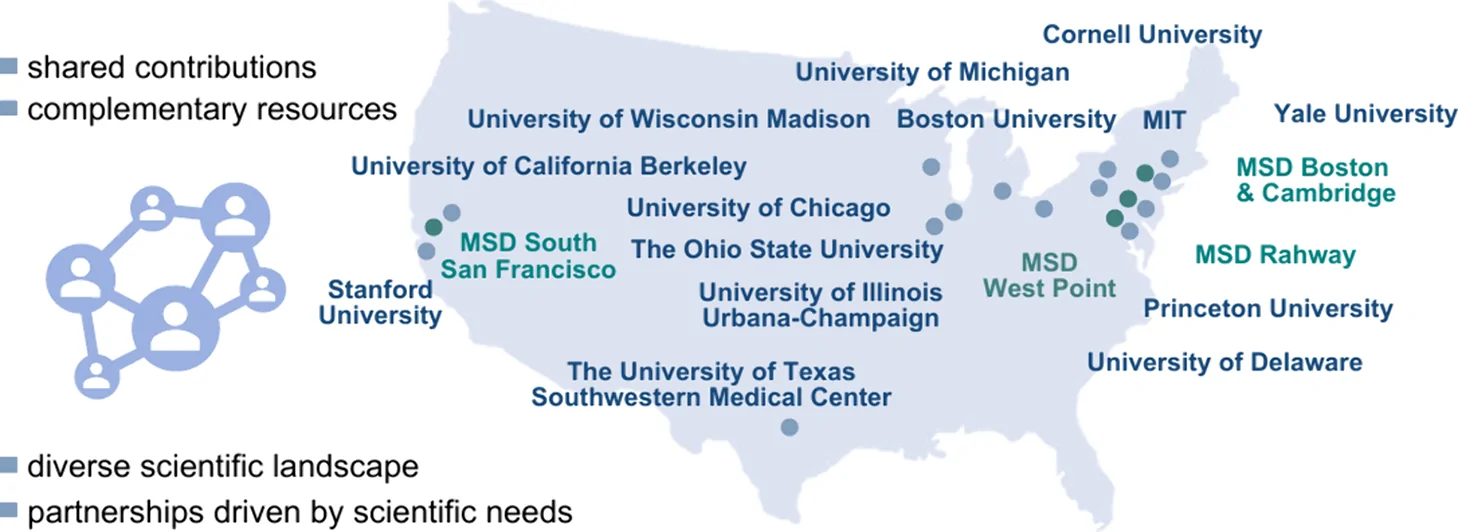

## 1 Introduction

Academic–industry partnerships are a long-standing catalyst for innovation in drug discovery, but their success depends on intentional alignment among all partners throughout the process.^1–6^ Impact is often measured as peer-reviewed publications or as methodological advances integrated into industrial drug discovery platforms. In this outlook, we highlight select MSD discovery chemistry collaborations disclosed over the past five years and use these case studies to articulate key characteristics for productive collaborations (Figure 1). Effective partnerships typically encompass four interdependent facets: (i) problem selection grounded in genuine scientific need; (ii) resource alignment, including people, infrastructure, data, and funding; (iii) disciplined project execution with clear communication of milestones; and (iv) impact generation via either publications or technology transfer. Academic and industry collaborators bring distinct expertise, and successful collaborations hinge on leveraging complementary strengths while continuously aligning on the project deliverables. MSD Discovery Chemistry (DC) collaborations that are synthesis-oriented largely fall within two distinct but complementary problem selection strategies. In the first strategy, the primary goal is methodological: to develop synthetic chemistry methods that solve specific bottlenecks in medicinal chemistry. The second strategy is centered around capability building: to create platforms and tools, such as high-throughput experimentation (HTE) capabilities or predictive computational models that can be applied broadly across reaction classes. Additionally, MSD DC extends beyond medicinal chemistry to include Modeling and Informatics (M&I), Protein and Structural Chemistry (PSC), and Chemical Biology groups. Reflecting the cross-functional nature of drug discovery, these teams also actively engage in a broad portfolio of collaborations that collectively accelerate drug discovery and development. The sections below describe collaborations across functions including synthetic chemistry, structural chemistry, M&I and chemical biology, and outline the academic and industrial resources central to their success. The outlook concludes with practical considerations for initiating new collaborations, sharing resources, maintaining effective communication, and extending impact beyond scientific advances.

**Figure 1:**
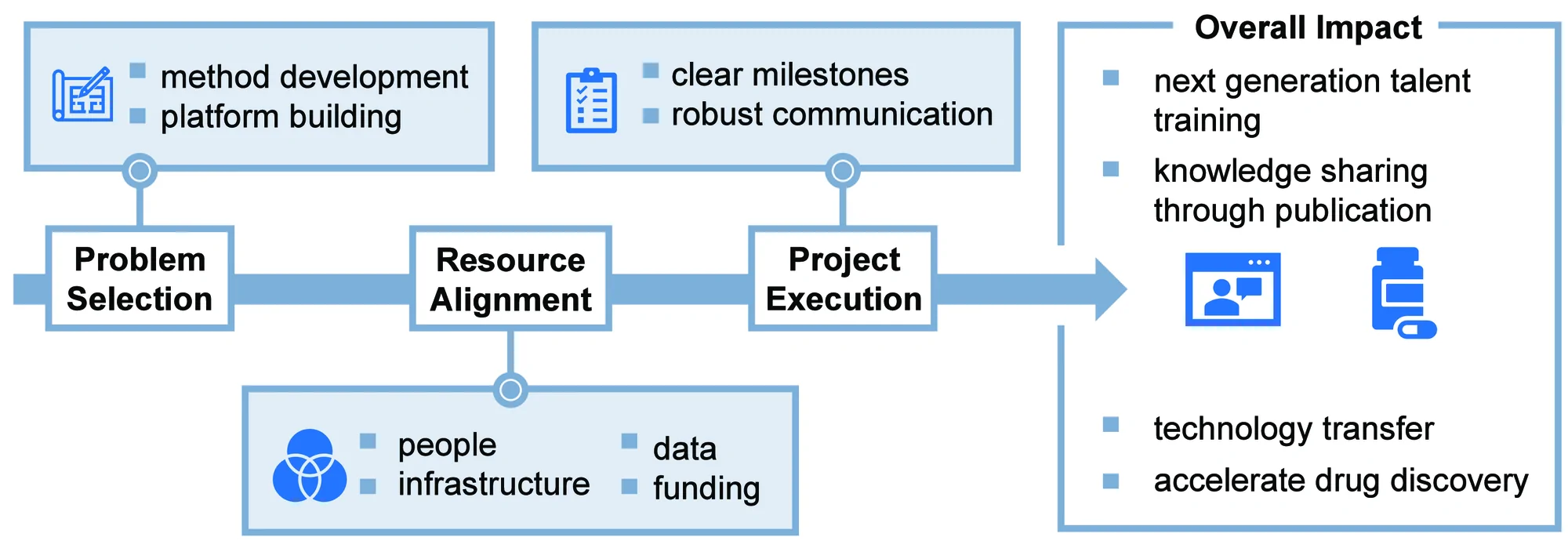
Framework for successful industry/academia collaborations in chemistry.

Academic–industry partnerships are a long-standing catalyst for innovation in drug discovery, but their success depends on intentional alignment among all partners throughout the process.

## 2 Small-Molecule Methodology

Efficient bond-forming reactions that operate reliably in complex molecular environments remain a persistent challenge in drug discovery. Accordingly, the development of new small-molecule synthetic methods is a central focus of many academia–industry collaborations in Discovery Chemistry. Specifically, synthetic methods that enable rapid access to diverse chemical space and are amenable to parallel syntheses are attractive. One recurring focus of these efforts is the synthesis of molecules with greater three-dimensional character, as sp^3^-rich structures are frequently associated with improved physicochemical and pharmacokinetic properties. Although MSD maintains a broad portfolio of academic partnerships in synthetic methodology.^7–11^ this section highlights a subset of collaborations focused on increasing molecular three-dimensionality. Rather than providing a comprehensive survey of recent efforts, these examples are presented to illustrate key facets that underpin productive collaborations.

### 2.1 Csp^2^–Csp^3^ Cross Couplings

In the context of medicinal chemistry, sp^2^–sp^3^ couplings are attractive for library synthesis due to the ready availability of diverse carboxylic acids and aryl halides.^12,13^ However, their scope is often limited, particularly with pharmaceutically relevant aryl halides under automation-amenable conditions.

To address these challenges, a collaboration between MSD and the MacMillan group at Princeton University adopted an atypical optimization strategy.^14^ Inspired by phenotypic screening in biology, the team conducted a “phenotypic” screen of aryl bromide–acid couplings in the presence of 721 distinct organic additives under otherwise standard conditions, leveraging MSD’s nanoscale HTE platform. This screen unexpectedly revealed that imides (e.g., phthalimide) significantly improved product yields relative to the additive-free reaction (Figure 2A). Further evaluation of structurally and electronically diverse imides identified phthalimide as the most broadly effective additive, enhancing yields across a wide range of aryl halide and carboxylic acid substrates. Mechanistic studies suggest that phthalimide stabilizes the Ni oxidative addition complex and suppresses undesired decomposition pathways, thereby promoting product formation. This work illustrates how empirical reaction optimization can both uncover superior conditions and deepen mechanistic understanding of synthetic transformations. Importantly, screening more than 700 additives was only practically feasible because of the nanoscale HTE capabilities at MSD.

**Figure 2:**
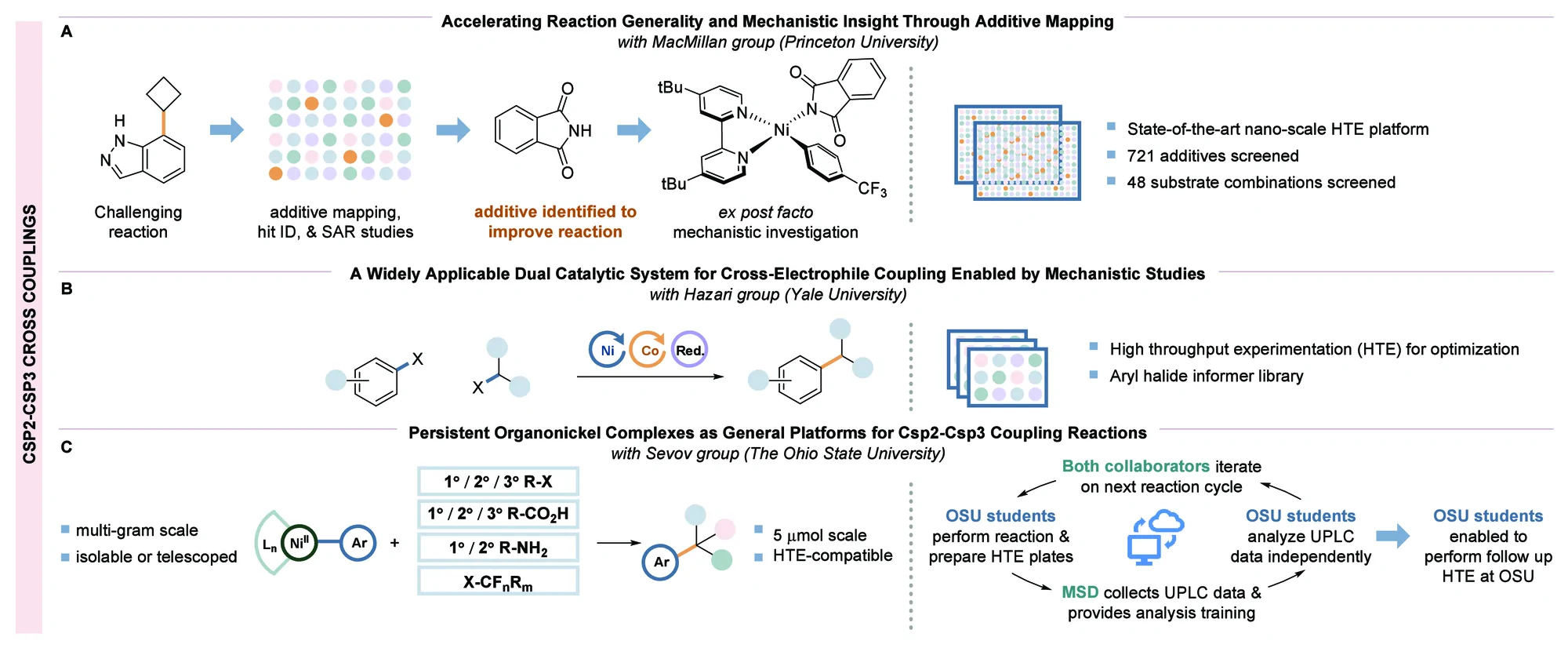
Collaborations focused on Csp^2^–Csp^3^ cross couplings. A) Accelerating reaction generality and mechanistic insight through additive mapping. B) A widely applicable dual catalytic system for cross-electrophile coupling enabled by mechanistic studies. C) Persistent organonickel complexes as general platforms for Csp^2^-Csp^3^ coupling reactions.

Cross-electrophile coupling (XEC) is another particularly attractive class of reactions, because it enables rapid analogue synthesis from commercially available aryl and alkyl electrophiles.^15^ The generally accepted mechanism for Ni-catalyzed XECs involves on-cycle organometallic Ni intermediates for both oxidative addition and alkyl radical generation, making it difficult to independently tune the rates of these steps. MSD’s collaborations with the Hazari and Sevov groups pursue complementary strategies to develop XECs that overcomes this challenge.

The MSD/Hazari group (Yale University) collaboration^16^ addressed this challenge using a dual-catalyst system in which each catalyst is responsible solely for oxidative addition or radical generation, thereby allowing greater control over these elementary steps. MSD’s high-throughput experimentation (HTE) capabilities, enabled rapid optimization across multiple substrate classes, including primary and secondary alkyl electrophiles as well as aryl iodides and bromides (Figure 2B). These couplings are also compatible with MSD’s informer aryl halide library,^17^ designed to pressure-test new methods using pharmaceutically relevant substrates. The Hazari group contributed deep mechanistic insight, while MSD chemists accelerated optimization and supplied drug-like substrates.

In a complementary XEC collaboration between MSD and the Sevov group at The Ohio State University (OSU), the aryl halide oxidative addition and radical generation are fully decoupled into two discrete steps, eliminating the need to balance their relative rates.^18,19^ Electrochemical reduction of (MeBPI)­NiII­(OAc) in the presence of aryl halides affords stable Ni oxidative addition complexes (OACs), which are subsequently reductively coupled with diverse alkyl electrophiles (Figure 2C). This approach was miniaturized to microscale (5 μmol in a 96-well HTE format), which is advantageous for medicinal chemistry campaigns that rely on precious starting materials and adhere to tight timelines. Subsequently, this platform was successfully extended to the more challenging sp^3^-sp^3^ cross-couplings leveraging couplings of Ni-alkyl oxidative addition complexes with diverse alkyl electrophiles.^18^ At the outset of the collaboration, Sevov group students from OSU visited MSD to conduct HTE experiments. As the work progressed, OSU students established HTE workflows in their own laboratory, running reactions at OSU and sending crude reaction mixtures to MSD for UPLC analyses. MSD-issued computers enabled the students to access and analyze the UPLC data remotely. This collaboration not only delivered a new method for small-molecule synthesis but also provided hands-on training in state-of-the-art HTE methods for students interested in industry careers.

### 2.2 Introducing BCPs to Increase Three-Dimensionality

The substitution of arene rings with bicyclo[1.1.1] pentanes (BCPs) in pharmaceutical candidates can improve properties such as solubility and metabolic stability.^20^ Although (1,3)-substituted BCPs are now widely used in medicinal chemistry, 2-substituted BCPs remain rare in the patent literature.^21^ Existing approaches often require long synthetic sequences and lack modularity, as the desired 2-substituent must be installed before construction of the BCP core.

The MSD collaboration with the MacMillan group enabled the development of a radical-mediated direct functionalization of bridge C–H bonds in 1,3-dicarboxylated BCPs to access the corresponding 2-brominated BCPs (Figure 3A).^22^ To accelerate downstream studies, MSD’s external research partner resources were used to prepare multigram quantities of this 2-brominated BCP, which served as linchpin intermediate for modular diversification via metallaphotoredox C–C and C–N cross-couplings. Several 2-substituted BCP analogues of known bioactive compounds were profiled at MSD (via external research partners) for potency, lipophilicity, solubility, and metabolic stability. Most BCP analogues retained potency relative to their aryl counterparts, and all tested BCPs displayed improved or comparable lipophilicity, solubility, and exposed polar surface area (EPSA) versus the corresponding 2-arylated or 2-aminated BCP drug molecules. Together, these results highlight the potential of 2-substituted BCPs as effective bioisosteres for *ortho*- or *meta*-substituted aryl rings in drug candidates.

**Figure 3:**
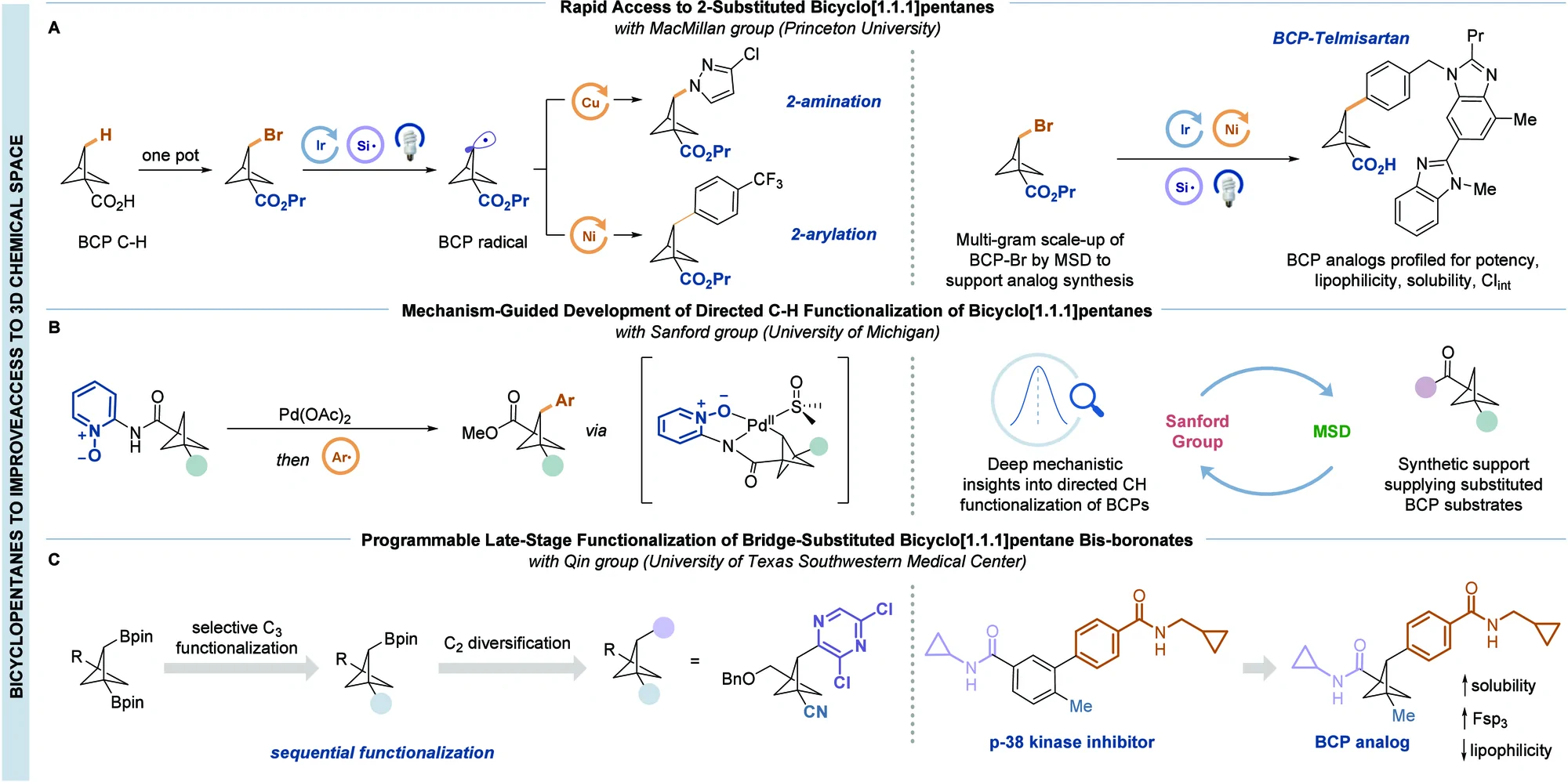
Method development to incorporate BCPs. A) Rapid access to 2-substituted bicyclo[1.1.1]­pentanes. B) Mechanism-guided development of directed C–H functionalization of bicyclo[1.1.1]­pentanes. C) Programmable late-stage functionalization of bridge-substituted bicyclo[1.1.1]­pentane bis-boronates.

The collaboration with the Sanford group from The University of Michigan focused on a complementary and previously challenging strategy to access 2-substituted BCP derivatives (Figure 3B).^23^ Sanford group’s expertise in mechanism-guided reaction development was leveraged to understand why earlier attempts at ligand directed BCP C–H functionalization had failed. Systematic experimental and computational studies were used to identify optimal directing groups and supporting ligands and to gain insight into the organometallic chemistry of BCPs. These insights were pivotal in developing a mild method to access cyclopalladated BCPs. A two-step sequence, BCP C–H activation followed by oxidative functionalization of the resulting palladacycles, provides a streamlined route to a key intermediate en route to Telmisartan analogues. This collaboration was enabled by MSD’s ability to supply substituted BCP substrates.

As this section’s final example of BCP functionalization, MSD and the Qin group from University of Texas Southwestern Medical Center developed a modular route to C1–C2 and C1,C2,C3-trisubstituted bicyclo[1.1.1]­pentanes (BCPs) that exploits the differential reactivity of bridgehead Bpin versus C2-Bpin units (Figure 3C).^24^ Bis-Bpin BCPs undergo selective C3 transformations (hydrazone alkylation, protodeborylation, C–X formation, Giese alkylation, cross-coupling), and subsequent manipulations furnish diverse C1–C2 and C1,C2,C3 substitution patterns. Replacement of arenes with these BCP motifs in drug-like scaffolds produced substantial solubility gains while retaining permeability; these studies were enabled by MSD’s experimental and analytical resources. Library enumeration using MSD’s virtual-library tools and principal moments of inertia (PMI) analysis^25^ showed that trisubstituted BCPs occupy a substantially broader 3D chemical space and are more sphere-like than corresponding 1,2,4- and 1,3,4-trisubstituted arenes. Notably, C1,C2-difunctionalized BCPs are more three-dimensional than analogous difunctionalized arenes and C1,C3-BCPs. These data establish C2-functionalization of BCPs as a strategic entry to novel, highly three-dimensional chemical space for medicinal chemistry.

### 2.3 C–H Functionalization

Methylene C–H bonds are often metabolic hotspots because they are susceptible to oxidation by liver enzymes. Identifying and characterizing the resulting metabolites is critical in drug discovery and frequently requires multistep de novo synthesis.^26^

The collaboration with the White group from University of Illinois Urbana–Champaign sought to leverage their expertise in C–H oxidation to streamline metabolite identification using Mn­(CF_3_–PDP) catalysis (Figure 4A).^27^ Under optimized conditions, several drug-like substrates underwent site-selective oxidation to afford products identical to those formed upon incubation with rat or human liver microsomes. A chemoinformatic analysis of the C–H oxidation reaction scope revealed that Mn­(CF_3_–PDP) catalysis significantly broadens the diversity of pharmaceutically relevant oxidized products relative to prior methods. In this collaboration, the White group conducted reaction optimization and scope studies, while MSD performed the rat and human liver microsomal assays. Collectively, the preparative nature of Mn­(CF_3_–PDP) small-molecule catalysis, combined with its chemo- and site-selectivity, establishes this platform as a functional mimic of CYP450 enzymes that can rapidly inform MetID and SAR in drug discovery campaigns.

**Figure 4:**
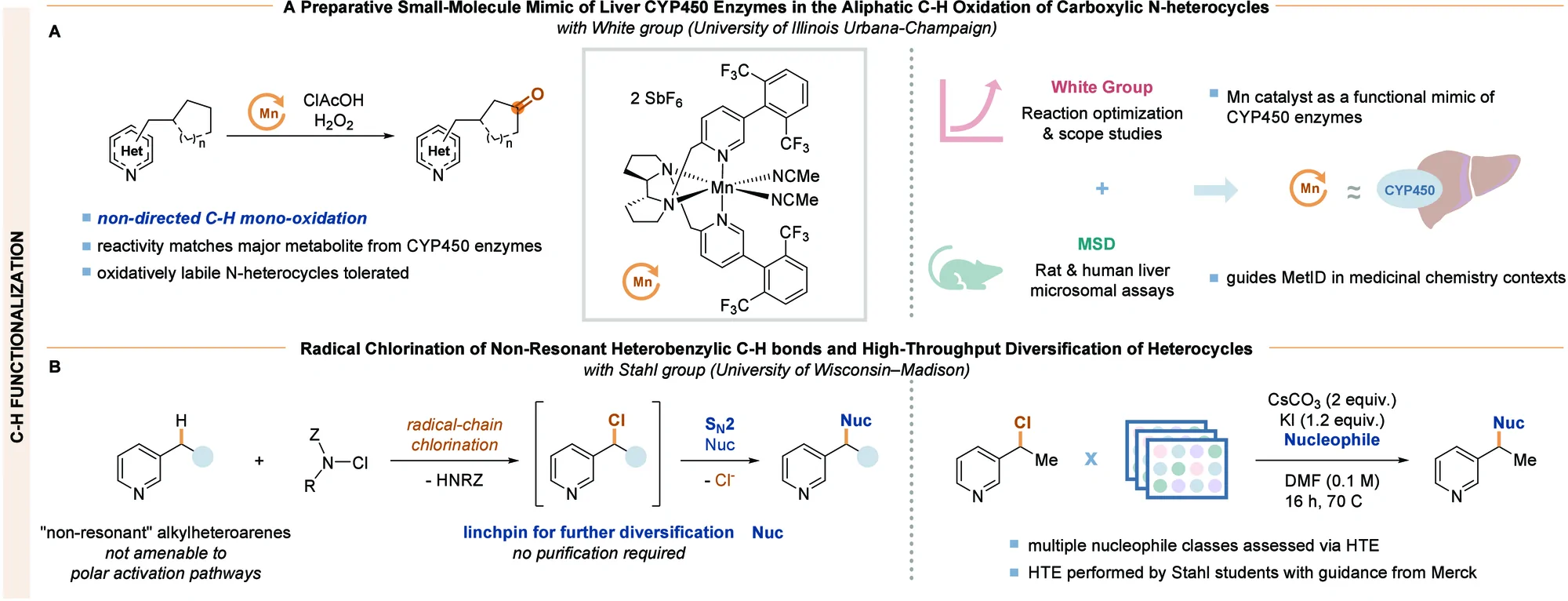
C–H functionalization methods. A) A preparative small-molecule mimic of liver CYP450 enzymes in the aliphatic C–H oxidation of carboxylic N-heterocycles. B) Radical chlorination of nonresonant heterobenzylic C–H bonds and high-throughput diversification of heterocycles.

Benzylic C–H bonds are common metabolic “hot spots,” and their selective replacement can markedly improve metabolic stability and other pharmacological properties. In collaboration with the Stahl group from University of Wisconsin-Madison, a series of Cu-catalyzed benzylic C–H functionalization methods were developed.^28,29^ Recent efforts have focused on tandem benzylic C–H functionalization/diversification sequences, in which an initial C–H functionalization generates a versatile linchpin that undergoes subsequent reactions with diverse nucleophiles (Figure 4B).^30^ Representative examples include (i) C–H chlorination followed by substitution with O, N, or C nucleophiles^31^ and (ii) C–H isocyanation followed by conversion of the isocyano group to ureas via reaction with amines.^32^ These transformations are applicable to benzylic C–H bonds in complex, pharmaceutically relevant substrates. In addition, the HTE-amenable displacement step enables parallel exploration of numerous nucleophiles, a key advantage for medicinal chemistry. Most HTE studies were carried out at MSD by Stahl group students, who gained first-hand experience in running HTE experiments and data analysis.

### 2.4 Non-Cross-Coupling Methods

Beyond the cross-couplings and C–H functionalizations described above, skeletal editing (i.e., targeted modification of a molecule’s core framework) has emerged as a powerful strategy in medicinal chemistry, enabling rapid access to previously inaccessible, drug-like chemical space and efficient lead diversification without *de novo* synthesis.^33^

In a long-standing collaboration with the Sarpong group from University of California, Berkeley, a Norrish Type II–like skeletal editing of six-membered saturated heterocycles to five-membered heterocycles and carbocycles was developed (Figure 5A).^34^ This transformation exhibits broad functional group tolerance and is applicable to the late-stage remodeling of pharmaceutical intermediates, peptides, and sugars. The Sarpong group contributed synthetic method development, while MSD provided medicinal-chemistry-relevant building blocks, NMR characterization, and computational support. The work was further enabled by an MSD-funded postdoctoral fellow,^35^ who conducted reaction optimization and substrate scope studies, integrating industrial resources with academically driven, pharmaceutically relevant research. The postdoctoral fellow also gained leadership experience by mentoring Sarpong group students.

**Figure 5:**
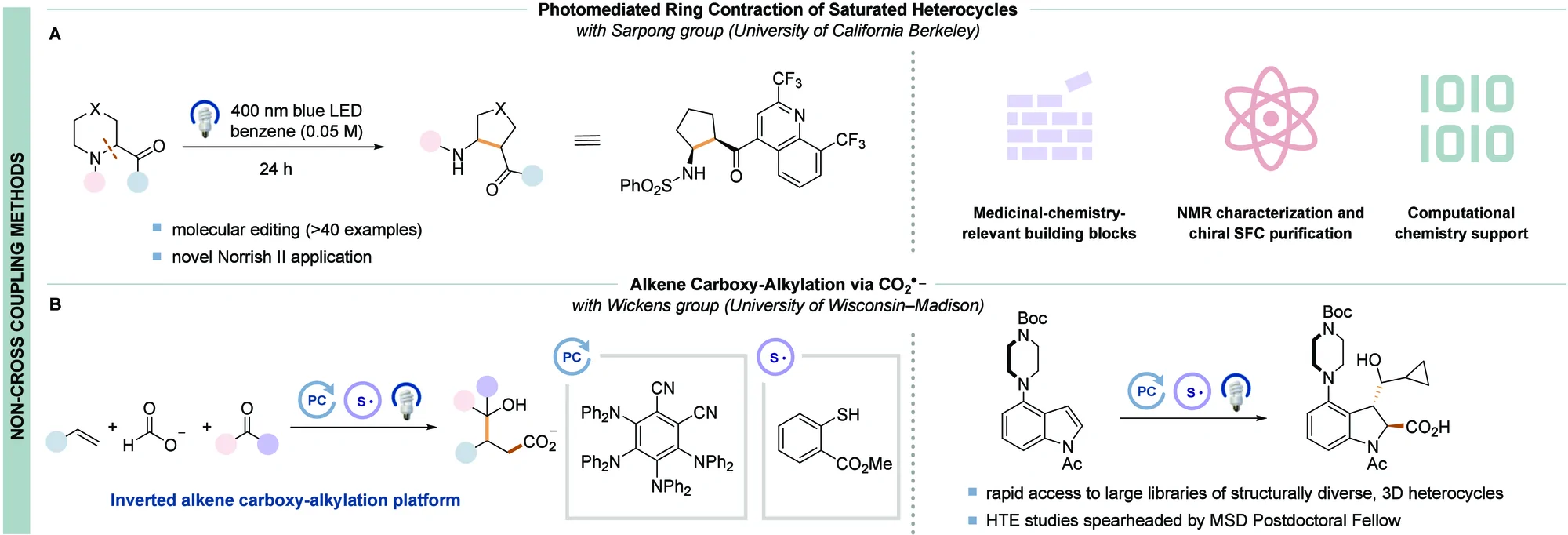
MSD-academia collaborations on noncross-coupling methods. A) Photomediated ring contraction of saturated heterocycles. B) Alkene carboxy-alkylation via CO_2_^•–^.

An effective complementary strategy for building 3D character is deplanarizing aromatic motifs via synthetic modification. In collaboration with the Wickens group from University of Wisconsin-Madison, a photoredox-mediated heteroarene hydrocarboxylation was developed that converts readily available planar heteroarenes into 3D analogues by introducing a single carboxylic acid vector (Figure 5B).^36^ This operationally simple transformation delivers versatile heterocyclic α-amino acids with broad scope and was subsequently implemented in HTE format on complex, drug-like indole-containing substrates.^37^ A 48-member heteroarene library, comprising building blocks to pharmacologically active compounds, underwent hydrocarboxylation with a ∼63% success rate (success defined as UPLC-MS assay yield >5%), and representative α-amino acid products were further diversified via HTE amenable amidation. This approach enables rapid access to large libraries of structurally diverse, 3D heterocycles through iterative hydrocarboxylation and diversification. Analogous to the Sarpong collaboration detailed above, an MSD postdoctoral fellow was involved and spearheaded the HTE studies for this collaboration.

## 3 Modalities beyond Small Molecules

Modern drug discovery has expanded beyond a purely small-molecule paradigm to incorporate peptides and antibody–drug conjugates (ADCs). From a medicinal chemistry perspective, these larger and more complex modalities demand new synthetic methods that can achieve site-selective modifications of densely functionalized substrates under mild conditions.

The collaboration between MSD and the M. Watson group from the University of Delaware developed a late-stage cross-electrophile coupling platform that enables the installation of diverse homoaryl alanines into complex macrocyclic peptides via alkyl Katritzky pyridinium intermediates (Figure 6A).^38^ The prominence of these noncanonical residues in peptide therapeutics has been well established, including enlicitide (MK-0616, MSD’s PCSK9 inhibitor in Phase 3 clinical trials for the treatment of hypercholesterolemia), LUNA-18, and chemical matter arising from a growing number of peptide drug discovery programs at MSD.^39–41^ At the same time, the availability of Fmoc-protected aryl and homoaryl alanine building blocks suitable for SPPS has remained limited. In light of these factors, a unified late-stage functionalization strategy was investigated using a common macrocyclic peptide alkylpyridinium precursor. Building on preliminary Watson group studies of aryl halide/Katritzky XECs, high-throughput experimentation at MSD was leveraged to overcome a key limitation of the original protocol: its incompatibility with protic functional groups such as carboxylic acids, which are common in drug discovery related macrocyclic peptides. The optimized conditions were showcased for the coupling of 3-bromo-5-phenylpyridine with a structurally advanced macrocyclic peptide intermediate sharing structural similarity to enlicitide (MK-0616). Although obtained in modest isolated yield (10%), the product was generated on a scale sufficient for initial *in vitro* potency assessment. This late-stage arylation platform is now leveraged to rapidly diversify macrocyclic peptides and accelerate peptide drug discovery programs at MSD.

**Figure 6:**
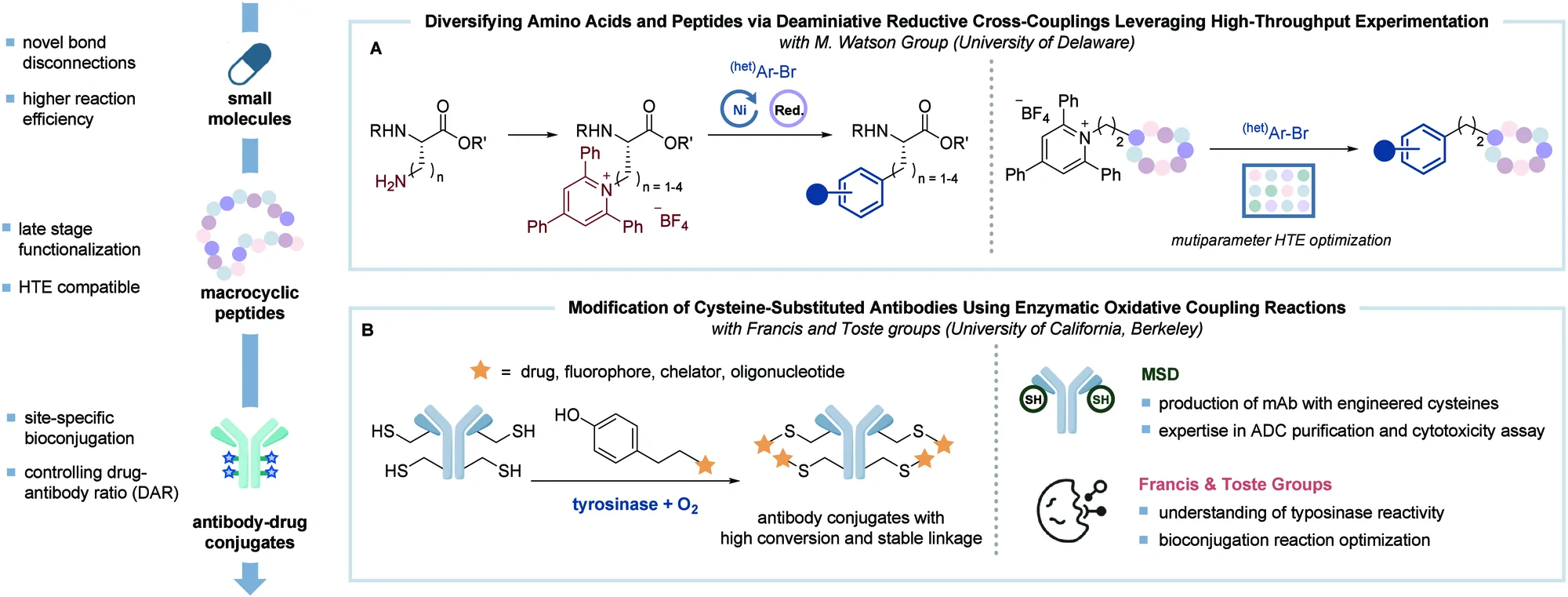
MSD-academia collaborations beyond small molecules. A) Diversifying amino acids and peptides via deaminiative reductive cross-couplings leveraging high-throughput experimentation. B) Modification of cysteine-substituted antibodies using enzymatic oxidative coupling reactions.

Mirroring the pharmaceutical industry’s growing interest in targeted chemotherapy such as antibody-drug conjugates (ADC), MSD’s academic collaborations extend into methodologies that furnish bioconjugates with increased efficiency and selectivity. One example is the application of tyrosinase-catalyzed phenol coupling with cysteine residues toward linker payload conjugation for ADC synthesis. The phenol/thiol reactivity was first reported by the Francis group from University of California, Berkeley in a protein–protein bioconjugation context, where the tyrosinase enzyme first promotes oxidation of the tyrosine phenol to a highly reactive *o*-quinone intermediate, which then undergoes nucleophilic attack by a cysteine thiol, producing a covalent C­(sp^2^)–S bond.^42–44^ In 2023, the Francis and Toste groups, in collaboration with MSD, reported an expanded application of this method, where linker payloads bearing phenols can undergo analogous tyrosinase-catalyzed oxidation to irreversibly react with cysteines engineered on antibody surfaces (Figure 6B).^45^ Using this method, the phenol-bearing cytotoxic monomethyl auristatin E (MMAE) payload was installed on HER2-targeting antibodies bearing engineered cysteines. The resulting ADCs with drug-to-antibody ratio (DAR) between 2 and 4 were evaluated in cytotoxicity assays to demonstrate that this enzymatic approach yields bioconjugates with good cell killing potential. MSD’s contributions to this work include the production of monoclonal antibodies bearing site-specifically engineered cysteines to enable controlled conjugation and well-defined DAR. In addition, MSD applied its established expertise in ADC purification and *in vitro* cytotoxicity assays to support evaluation of conjugate quality and functional potency in relevant cell-based models.

## 4 Capability Build Collaborations

In addition to academic collaborations that enable synthetic methodology development, another important goal of MSD’s collaborations surrounds drug-discovery relevant capability building. These efforts center on the creation or advancement of enabling technologies, including innovations in reactor and instrumentation design and the development of machine learning *in silico* models for reactivity prediction. Collectively, such collaborations lead to developing new or improving existing platforms to accelerate the drug discovery process.

The first capability-build example highlighted herein is HTe–Chem, a microscale, high-throughput 24-well electrochemical reactor developed through collaboration between the Lin group from Cornell University and Process Research & Development and Discovery Chemistry groups at MSD with key design and build support from MSD’s Scientific Engineering group (Figure 7A).^46^ Electrochemistry can offer reactivity complementary to thermal and photochemical methods, enabling access to otherwise inaccessible scaffolds for drug discovery. HTe–Chem represents a substantial advance in high-throughput electrochemistry: it is compatible with ANSI/SLAS microplate standards and supports constant-current, constant-voltage, and electrophotochemical screening. Integration with the MSD Engineering division was pivotal to rapidly accessing and iterating the reactor prototypes. Since its initial disclosure and commercialization, HTe–Chem has been applied to reaction-optimization campaigns in both industrial and academic settings, including the development of electrochemical reductive couplings of alkylpyridiniums.^47^ In these studies, HTe–Chem enabled efficient multiparameter optimization and rapid evaluation of reaction scope. The collaboration leveraged the Lin group’s electrochemical expertise, with cross-site reproducibility rigorously established through parallel validation by Lin group researchers and MSD scientists, with validation at MSD being led by an MSD postdoctoral fellow.

**Figure 7:**
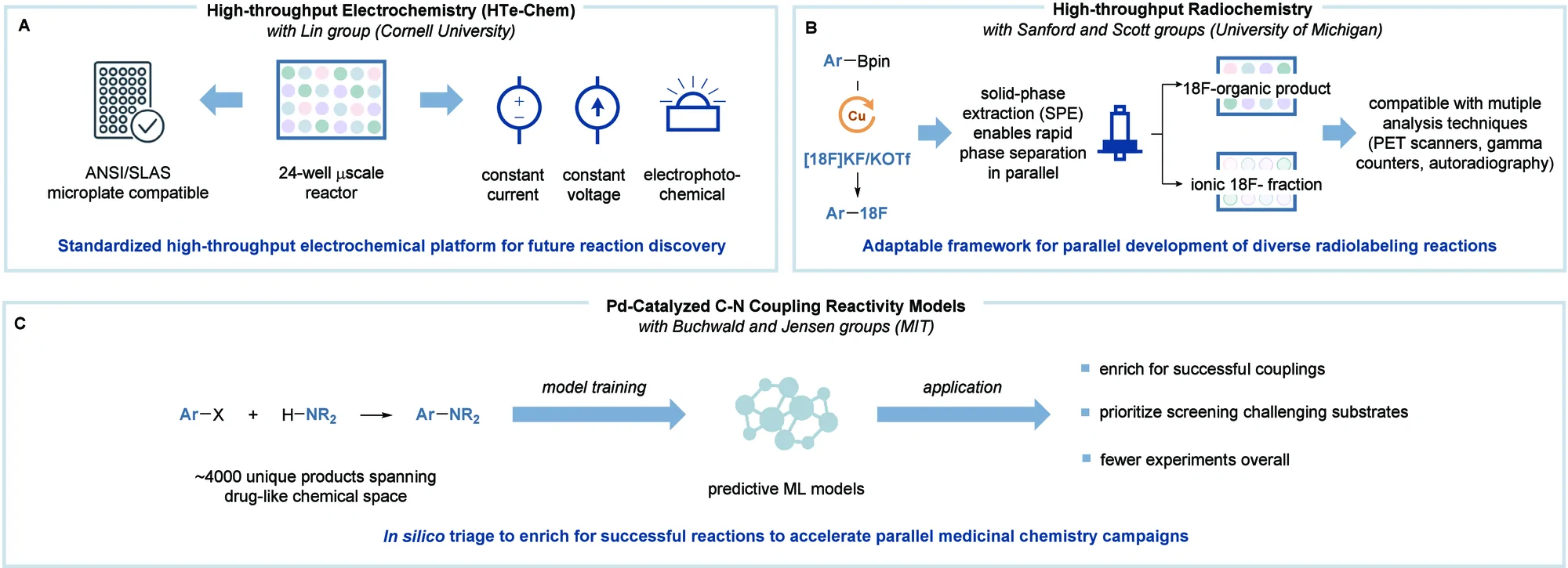
Building discovery capabilities through MSD-academia collaborations. A) High-throughput electrochemistry (HT*e* ^*–*^ Chem). B) High-throughput radiochemistry. C) Pd-catalyzed C–N coupling reactivity models.

In collaboration with the Sanford and Scott groups from The University of Michigan, MSD has developed a high-throughput radiochemistry platform for copper-mediated radiofluorination (CMRF) of (hetero)­aryl boronate esters using commercial 96-well reaction blocks combined with plate-based solid-phase extraction (SPE) (Figure 7B).^48^ Reactor design, supported by MSD’s engineering division, was pivotal to achieving robustness and reproducibility across the full plate. The SPE workflow enables rapid, parallel separation of aqueous and organic radioactive fractions, effectively replacing radio-TLC with improved speed, throughput, and operational simplicity. Coupled with rapid, parallel analytical readouts, including PET-based imaging, gamma scintillation counting, and autoradiography, the platform enables quantitative assessment of radiochemical conversion across 96 reactions in a single experiment. This capability was applicable to the systematic lead optimization of drug-like boronate esters, and directly translatable to conventional radiochemistry performed at approximately 10-fold larger scale. To ensure robustness and transferability, the workflow was implemented and validated both in academic laboratories and in MSD radiochemistry facilities. Overall, this work establishes a general, high-throughput framework for radiofluorination that accelerates reaction discovery and optimization while simultaneously reducing radiation exposure. The platform is simple, economical, and built from commercially available equipment, facilitating broad adoption and standardization of workflows in radiochemistry laboratories. Although demonstrated here in a 96-well format, the approach is readily extendable to higher-density plates (384–1536 wells). More broadly, the conceptual framework developed for this high-throughput radiochemistry platform is adaptable beyond radiofluorination, offering a blueprint for parallelized development of diverse radiolabeling transformations.

High-throughput experimentation (HTE) is widely used to optimize reaction conditions, but exhaustive multiparameter screening is time- and resource-intensive, especially in medicinal chemistry where large analogue libraries are needed. A recent collaboration with the Buchwald and Jensen groups from Massachusetts Institute of Technology established a forward-looking workflow that uses predictive machine learning models to prospectively assess reaction performance *in silico* and prioritize only challenging substrates for experimental screening (Figure 7C).^49^ Pharmaceutically relevant predictive models were built for Pd-catalyzed C–N cross-coupling using MSD’s building block collection to select hundreds of aryl halides and amines. MSD nanoscale HTE capabilities enabled rapid generation of ∼4,000 unique products, yielding a data set with high structural complexity, rich functional group diversity, and strong overlap with drug-like chemical space. The large data set enabled systematic evaluation across five data-splitting strategies rigorously testing model interpolation and extrapolation. The resulting classification models significantly enriched successful C–N couplings when used prospectively, enabling high-fidelity *in silico* triage of reaction conditions and substrates. This generalizable workflow lays the foundation for predictive modeling across diverse reaction classes to enable data-driven HTE campaigns. With respect to resource-sharing, machine learning models and associated data science workflows were developed by the academic scientists while MSD enabled the generation of the data set for modeling. A MSD postdoctoral fellow joined the collaboration to enhance industrial bandwidth, advancing the project through both experimental execution and manuscript compilation by assuming a leadership role in coordinating with academic trainees.

## 5 Discovery Chemistry Collaborations beyond Synthesis

The collaborations described above have primarily focused on accelerating the design–make–test-analyze cycle through the development of new synthetic methods, increasing synthetic throughput via HTE, or enabling prioritization of molecules for synthesis. Beyond these efforts, as described below, MSD’s Discovery Chemistry organization has also pursued strategic collaborations aimed at ADMET (absorption, distribution, metabolism, excretion, toxicity) property prediction, target identification and biological mechanism-of-action elucidation.

Beyond reactivity predictions described above, equally important are predicting ADMET properties of drug molecule designs before their synthesis. ADMET properties are key determinants of drug efficacy and safety, making their accurate prediction a central challenge in drug discovery. Recognizing the limitations of existing approaches, the Modeling and Informatics (M&I) division within MSD Discovery Chemistry led a systematic effort to advance ADMET modeling through a close academic-industry collaboration with the Pande lab at Stanford University (Figure 8A).^50^ Together, the teams codesigned and evaluated modern deep-learning strategies for ADMET prediction, culminating in the development of a multitask variant of PotentialNet. A Pande lab graduate student joined MSD as a part-time contractor, which enabled them to access MSD computing resources and proprietary compound data for model training and validation. MSD defined rigorous, prospective benchmarking criteria, including temporal and extrapolative test splits, to reflect real-world medicinal chemistry use cases. Model performances were evaluated across 31 MSD ADMET end points, including LogD, solubility, permeability, and other pharmacokinetic properties. Under these conditions, the multitask PotentialNet consistently outperformed MSD’s descriptor-based random forest baselines. The models were successfully piloted on active MSD discovery programs, providing proof-of-concept in realistic settings. These results informed MSD’s internal development of a related multitask graph neural network framework, which has since been deployed at scale and is now used across MSD chemistry teams to guide lead optimization.^51^

**Figure 8:**
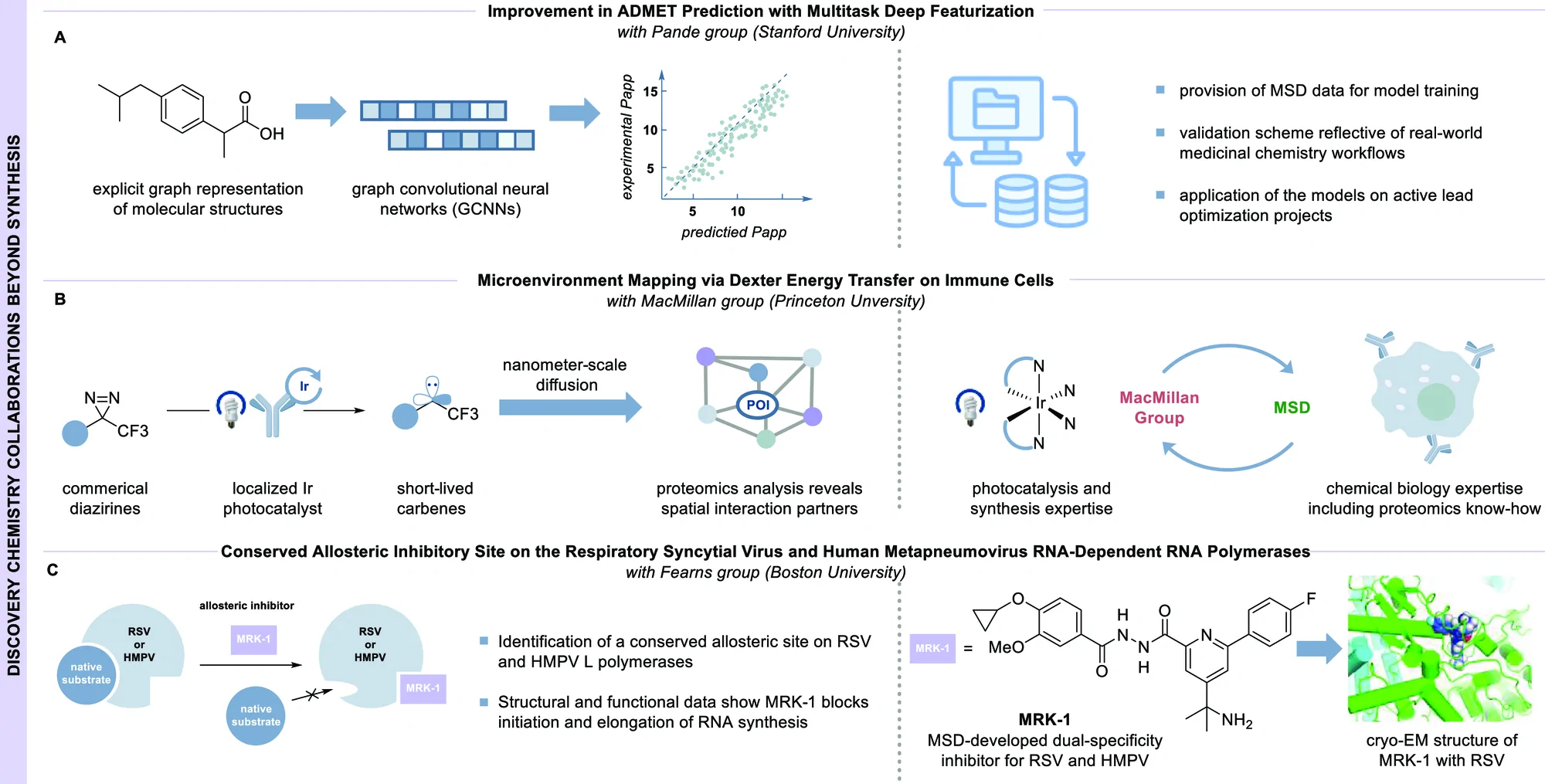
MSD discovery chemistry collaborations beyond synthesis. A) Improvement in ADMET prediction with multitask deep featurization. B) Microenvironment mapping via Dexter energy transfer on immune cells. C) Conserved allosteric inhibitory site on the respiratory syncytial virus and human metapneumovirus RNA-dependent RNA polymerases.

The Chemical Biology division within MSD Discovery Chemistry led a collaboration with the MacMillan Group that combined MSD’s chemical biology expertise with the MacMillan Group’s strengths in photocatalysis to develop antibody-directed proximity and photoaffinity labeling methods. The resulting method, termed MicroMap (μMap), leverages blue light photoactivation of diazirines using ligand-tethered iridium photocatalysts for local generation of short-lived carbene intermediates (Figure 8B). These carbene intermediates diffuse only a few nanometers before reacting with neighboring biomolecules, tagging them for downstream proteomics identification. In 2020, the team described application of μMap as a high-resolution proximity labeling technology for elucidation of a disease target’s spatial interaction network.^52^ Here, the authors attached an Ir-polypyridyl photocatalyst to the programmed-death ligand 1 (PD-L1) antibody, which selectively binds to PD-1 antigens on the cell surface, thereby uncovering known and novel interactors of PD-1. In 2022, the team described application of μMap to demonstrate that iridium conjugates of small molecule drugs can be used to tag surface and intracellular proteins as a powerful new target identification method.^53^ Using iridium conjugates of small molecule drugs including paclitaxel (Taxol), dasatinib (Sprycel), and ligands for GPR40, A2A and BRD4, demonstrated superiority of catalytic carbene generation and resulting signal amplification, over traditional photoaffinity labeling approaches which rely on stochiometric carbene generation. Born from collaborative brainstorming sessions and enhanced by a productive cross-training visit of academic scientists to MSD, the μMap invention stands as a fantastic example of exciting science that results from synergies inherent to industry-academic collaboration. Importantly, not only did this effort bolster MSD’s chemical biology toolbox, but it also yielded a rapid expansion of photocatalytic protein labeling methods and additional novel applications of the technology.

The Protein and Structural Chemistry (PSC) division within MSD Discovery Chemistry contributed to a collaboration with the Fearns Group from Boston University, whose research focuses on the transcriptional and replication mechanisms of nonsegmented negative-strand RNA viruses, including respiratory syncytial virus (RSV) and human metapneumovirus (HMPV) (Figure 8C).^54^ Although small-molecule inhibitors targeting the RSV polymerase are under development, their binding sites and molecular mechanisms of action have remained largely unknown. MSD’s PSC group contributed to addressing this gap by determining cryo-EM structures of RSV and HMPV polymerases in complex with inhibitors, revealing key inhibitor–polymerase interactions and identifying specific protein–ligand contacts that can be potentially guide future medicinal chemistry efforts for the rational design of next-generation pneumovirus polymerase inhibitors. Furthermore, integration of these structural insights with complementary chemical biology assays performed at the Fearns lab was pivotal to understanding the underlying mechanism of action of polymerase inhibition. Overall, this collaboration was highly efficient because the Fearns Lab already possessed specialized RSV polymerase assays and mechanistic expertise; in return, MSD supplied high-quality small-molecule inhibitors and cryo-EM data.

## 6 Initiating Collaborations

We hope these examples encourage academic and industry partners to pursue new collaborations, often initiated through informal exchange of ideas that explore shared scientific interests. When interests converge, industry partners typically invite a formal proposal for consideration. At MSD, any scientist, not just those in specialized capability groups, can initiate collaboration proposals, reflecting a democratized approach. Following proposal approval, a legal agreement is established to govern resource sharing, a process that in general can take several months to a year.

At MSD, any scientist, not just those in specialized capability groups, can initiate collaboration proposals, reflecting a democratized approach.

Collaboration contracts are generally structured for 2–3 years, with opportunities for renewal by mutual agreement. Contracts may be established with individual PIs or with entire university departments (i.e., master agreements). In the latter case, follow-up contractual agreements with individual academic PIs are often expedited. Students visiting MSD may also require a supplemental “visiting scientist” agreement. Support for academic partners can be provided in kind (e.g., materials or access to infrastructure) or as a combination of in-kind resources and financial support, depending on project needs and budgets.

## 7 Resource Alignment and Communication

Successful academia–industry collaborations hinge on strategically leveraging complementary strengths and resources from both partners. This coordination between academic and industry collaborators begins as early as problem selection and continues throughout the project, often realigning as the science evolves with data. During problem selection, academic collaborators contribute subject-matter expertise within their respective subfields, which could take the form of deep mechanistic insight or conceptual innovation. On the other hand, the industrial collaborators at MSD offer perspectives on how a project might directly accelerate medicinal chemistry campaigns, keeping the collaboration focused on pharmaceutical relevance. For synthesis-oriented collaboration, these considerations may include identifying substrates with realistic pharmaceutical complexity to guide new methodology development or defining engineering requirements for new reactors that are practical for wide adoption by drug discovery practitioners. For nonsynthesis-oriented collaborations, industry insight is often focused on identifying unmet needs in tools for drug development, such as more accurate *in silico* predictions of molecular properties or better understanding of the biological pathway of a disease target of interest.

During project execution, industrial collaborators accelerate progress through access to high-throughput experimentation, analytical infrastructure, and extensive building-block collections. MSD’s extensive compound collection also enables efforts beyond synthetic chemistry collaborations, such as the generation of large, pharmaceutically relevant data sets for ADMET ML models. Academic partners often complement these resources by engaging dedicated graduate students and/or postdoctoral fellows, whose focused efforts allow them to progress key scientific deliverables. On the other hand, industrial collaborators can expand their bandwidth by hiring MSD-funded postdoctoral fellows who are focused on driving the collaboration forward. In many cases, academic researchers on a collaboration may visit MSD laboratories for cross-training.

Successful academia–industry collaborations are grounded in deliberate, transparent communication that links industrial principal investigators (PIs), academic PIs, the trainees, and the scientists executing the work. This often takes the form of regular meetings (frequently virtually) to update project progress. Active engagement from PIs on both sides is critical for securing resources, aligning expectations, and sustaining project momentum. Equally important is intentional investment in graduate student and postdoctoral fellow development, ensuring that collaborations contribute meaningfully to their professional growth. Trust, responsive communication, and a shared commitment to problem-solving collectively determine a collaboration’s ability to adapt to the inevitable challenges inherent to scientific research.

Through publications and conference presentations, these partnerships amplify visibility for all collaborators by highlighting shared scientific achievements.

## 8 Impact beyond Science

Beyond scientific advances, these collaborations foster professional development across academic and industrial settings. Academic trainees benefit from exposure to both academic and industrial perspectives, gaining insight into how fundamental chemistry translates into drug discovery while acquiring hands-on experience with industrial high-throughput experimentation (HTE), advanced analytical platforms, engineering approaches, and data science workflows. Conversely, industrial trainees, such as MSD postdoctoral fellows, gain mentorship and leadership experience through the integration of industrial capabilities with academically driven, pharmaceutically relevant research. Additionally, industrial PIs strengthen their leadership skills by defining the scientific goals of the project and mentoring the trainees in executing those objectives. Through publications and conference presentations, these partnerships amplify visibility for all collaborators by highlighting shared scientific achievements. Finally, these partnerships provide visibility into academic talent pools, thereby expanding and strengthening MSD’s pipeline for next-generation scientific talent.

. . .these partnerships provide visibility into academic talent pools, thereby expanding and strengthening MSD’s pipeline for next-generation scientific talent.

## 9 Summary

This outlook surveys MSD Discovery Chemistry collaborations from the past five years to distill practical principles for productive academia–industry partnerships in drug discovery. Using representative case studies, it highlights two complementary collaboration models: targeted development of synthetic methodologies that address concrete medicinal chemistry bottlenecks, and capability-building efforts that enable technology transfer. Furthermore, a few nonsynthetic chemistry collaborations are also detailed highlighting the cross-functional nature of discovery chemistry. These partnerships leverage complementary strengths from each collaborator: academic insight and innovation paired with industrial perspectives and resources.

We hope this outlook inspires both academic and industrial chemists to pursue collaborations that are well aligned in research interests and rooted in medicinal chemistry relevance, ultimately accelerating drug discovery and accelerating the delivery of therapeutics to patients in need.

The case studies illustrate how intentional problem selection, disciplined project execution, and robust resource sharing enable both high-impact publications and direct integration of new technologies into medicinal chemistry practice. Beyond scientific outcomes, collaborations enhance trainee development through direct exposure to both academic and industrial research environments. Sustained communication, trust, and alignment on impact are critical. We hope this outlook inspires both academic and industrial chemists to pursue collaborations that are well aligned in research interests and rooted in medicinal chemistry relevance, ultimately accelerating drug discovery and the delivery of therapeutics to patients in need.
